# Attitudes toward tobacco-free and cannabis-free policies among residents in permanent supportive housing who use tobacco, cannabis, and other substances

**DOI:** 10.1016/j.abrep.2026.100686

**Published:** 2026-03-10

**Authors:** Narges Neyazi, Deepalika Chakravarty, Fan Xia, Mark R. Hawes, Wendy Max, Margot Kushel, Maya Vijayaraghavan

**Affiliations:** a Center for Tobacco Control Research and Education, University of California, San Francisco, CA, USA; b Center for AIDS Prevention Studies, Division of Prevention Science, University of California, San Francisco, CA, USA; c Department of Epidemiology and Biostatistics, University of California, San Francisco, CA, USA; d Department of Social and Behavioral Sciences, University of California, Max, San Francisco, CA, USA; e Institute for Health & Aging, University of California, San Francisco, CA, USA; f Division of Health Equity and Society, University of California, San Francisco, CA, USA; h Division of General Internal Medicine, San Francisco General Hospital, University of California, San Francisco, CA, USA; i UCSF Division of General Internal Medicine, University of California, San Francisco, CA, USA

**Keywords:** Tobacco use, Cannabis use, Attitudes towards smoke-free policies, Permanent Supportive Housing, Tobacco and cannabis co-use, Substance use

## Abstract

**Background:**

Over 60% of permanent supportive housing (PSH) residents smoke cigarettes, and over 50% use cannabis. Co-use of tobacco and cannabis may be linked with other substance use. We explored attitudes toward tobacco-free and cannabis-free policies among PSH residents who smoked cigarettes, co-used tobacco and cannabis, or other substances.

**Methods:**

Between 2022 and 2024, we recruited 400 PSH residents who smoked cigarettes into a cluster-randomized clinical trial (RCT) of a smoke-free home intervention. Using baseline data from the RCT, we created scores reflecting attitudes toward tobacco-free and cannabis-free policies (higher scores reflect more favorable attitudes). We used linear mixed models to examine multivariable associations of these attitudes with predisposing, enabling, and need factors, as well as with intensity of tobacco and cannabis co-use.

**Results:**

Participants’ mean age was 54.5 years, 62.7% were male, and 41.8% identified as Black or African American. Of the participants, 64.3% used cannabis, 31.5% used amphetamines, and 27.3% used cocaine in the past 30 days. Cocaine use in the past 30 days was associated with less favorable attitudes toward tobacco-free policies (adjusted β: −0.20, 95% CI: −0.37, −0.04). Compared to light co-users of tobacco and cannabis, heavy co-users of tobacco and cannabis had less favorable attitudes toward tobacco-free policies **(**adjusted β**:**-0.32, 95% CI: −0.56, −0.07) and cannabis-free policies (adjusted β:-0.58; 95% CI: −0.82,-0.35).

**Conclusions:**

Interventions that address the intensity of tobacco and cannabis co-use, the high levels of indoor co-use of tobacco and cannabis, and co-occurring substance use may increase favorable attitudes towards smoke-free policies in PSH.

## Highlights

• Two-thirds of study participants in permanent supportive housing (PSH) co-used tobacco and cannabis.
• Over half of the tobacco and cannabis co-users reported past-30-day other substance use.
• Heavy tobacco and heavy cannabis co-users had less favorable attitudes toward smoke-free policies.
• Past 30-day cocaine use was associated with less favorable attitudes toward tobacco-free policies.
• Interventions that address tobacco and cannabis co-use and other substance use are needed.

## 1 Introduction

Tobacco use is the leading preventable cause of death in the United States, contributing to over 490,000 deaths annually (US Department of Health and Human Services, 2024). Tobacco use disproportionately impacts adults living in permanent supportive housing (PSH). PSH is subsidized housing with voluntary on-site or closely linked services for adults experiencing chronic homelessness with a qualifying disability that includes mental health and substance use disorders (Tsemberis et al., 2004).

An estimated 60% of adults living in PSH report current smoking (Petersen et al., 2018), and a third report using other forms of combustible tobacco (Durazo et al., 2020). An estimated 53% of tobacco users living in PSH also use cannabis (Durazo et al., 2020). The high rates of secondhand smoke (SHS) exposure and chronic disease linked with tobacco and cannabis use among adults living in PSH (Bero et al., 2023) underscore the importance of smoke-free housing that promotes health and overall well-being (Henwood et al., 2013; Henwood et al., 2015, Vijayaraghavan & King, 2020).

In 2018, the Department of Housing and Urban Development implemented smoke-free policies in public housing; however, these policies did not apply to multi-unit PSH (United States: Housing and Urban Development Department: Multi-Family Housing Office, 2014). Nationally, 24 states and two territories have legalized cannabis for recreational use (National Conference of State Legislatures, 2024). Although there is a movement towards implementing smoke-free policies in multi-unit housing (Goldberg and Levy, 2023, Lathen et al., 2020), such policies have faced implementation challenges in PSH because of concerns about increasing evictions and homelessness due to policy violations, and also the legalization of cannabis (Alizaga et al., 2019, Vijayaraghavan and King, 2020).

Studies have shown that residents support a tobacco-free lifestyle in multi-unit public housing and PSH (Lee et al., 2022, Vijayaraghavan et al., 2025). Residents in Wisconsin’s public housing had favorable attitudes toward a tobacco-free policy, although people who smoked had more negative attitudes than those who did not smoke (Lathen et al., 2020). The policy also had a positive impact on health, with over 80% reporting at least one change in their smoking, including attempting to quit, reducing smoking, calling the quitline, or using quit aids, and 6% reporting that they were motivated to quit because of the policy (Lathen et al., 2020). Among PSH residents in California who lived in a building that adopted smoke-free policies, residents’ attitudes toward smoke-free policies improved, and the prevalence of smoking decreased after smoke-free policy implementation (Petersen et al., 2018).

There are mixed views on support for cannabis-free policies in multi-unit housing (Petersen et al., 2020, Vijayaraghavan et al., 2025). In a nationally representative sample of adults, stronger perceptions of harm from secondhand cannabis smoke were associated with a higher likelihood of a home cannabis smoking policy (Tripathi et al., 2024). However, only a minority of the sample perceived cannabis to be harmful (Tripathi et al., 2024). In another nationally representative sample of residents living in multi-unit housing, individuals who smoked cigarettes were more likely to allow smoking and vaping indoors (nicotine and cannabis) and were less likely to support rules around smoking indoors compared to residents who did not smoke (Reyes-Guzman et al., 2023).

Although these studies have examined attitudes toward indoor use of tobacco or cannabis among adults living in multi-unit housing, no studies have examined such attitudes among co-users of tobacco and cannabis. Neither are there studies that examine attitudes toward indoor tobacco-free and cannabis-free policies based on the intensity of co-use or use of other substances. Given the increasing prevalence of tobacco and cannabis co-use among PSH residents, our study fills an important gap in the field by examining: 1) attitudes toward tobacco-free policies among adults who report smoking tobacco indoors in PSH; 2) the prevalence and intensity of tobacco and cannabis co-use, and 3) attitudes toward tobacco-free and cannabis-free policies among tobacco and cannabis co-users as well as people who use other substances. We hypothesized that heavy tobacco and heavy cannabis co-users would have less favorable attitudes towards tobacco-free and cannabis-free policies.

## 2 Methods

We used baseline data from an ongoing cluster-randomized clinical trial (RCT) of an intervention to increase voluntary adoption of smoke-free homes among residents living in PSH in the San Francisco Bay Area. The study protocol is published (Odes et al., 2022), and the trial is registered on clinicaltrials.gov (NCT04855357) (Vijayaraghavan, 2025). The University of California, San Francisco Institutional Review Board approved and monitored all study procedures (IRB # 20–33214), and all participants signed an informed consent form. Participants received a $20 gift card for completing the baseline questionnaire.

### 2.1 Participants and Setting

We recruited 400 adults from 40 PSH sites in the San Francisco Bay Area. PSH sites were single-site, multi-unit housing with optional on-site case management and other social services (Henwood et al., 2024). Eligible participants were (1) residents of the PSH site and planned to live there for at least 12 months, (2) 18 years or older, (3) English-speaking, (4) able to provide informed consent, (5) currently smoking cigarettes (at least five cigarettes per day in the past 7 days, verified by expired carbon monoxide,≥ 8 ppm) (Benowitz et al., 2002) and (f) currently smoking in their homes.

### 2.2 Theoretical framework

We applied the Andersen-Gelberg Model for Vulnerable Populations (Gelberg et al., 2000), initially developed to understand health services use and health outcomes among vulnerable populations, to evaluate predisposing, enabling, and need factors associated with attitudes toward tobacco-free and cannabis-free policies in PSH. Predisposing factors include demographic characteristics prior to the need for services; enabling factors include structural resources, such as insurance, that facilitate access to services; and need factors include more direct causes of needing services, such as mental health or substance use. If predisposing, need, and enabling factors were associated with attitudes toward indoor tobacco-free and cannabis-free policies, then those same factors may be associated with health behaviors like adopting a smoke-free home.

### 2.3 Outcome variables

We evaluated two outcome variables: attitudes toward tobacco-free policies and attitudes toward cannabis-free policies. We used our previously developed tobacco-free attitudes scale (Durazo et al., 2020, Petersen et al., 2018, Petersen et al., 2020, Vijayaraghavan and Pierce, 2015) and adapted it for cannabis-free attitudes. We asked participants to report their level of agreement with a hypothetical tobacco-free policy and cannabis-free policy that restricted smoking tobacco or cannabis, respectively, in indoor living areas. Participants responded to the following five statements for tobacco and cannabis use: 1) I would support such a policy, 2) I would try to move to another property that allows smoking indoors, 3) I would try to cut down on my smoking because of the policy, 4) I would try to stop smoking completely because of the policy, and 5) I would continue to smoke inside my apartment. The cannabis-free attitude items had an additional response option of “does not apply” for those who were not current users of cannabis. For the cannabis attitudes scale, only participants who answered at least 4 of the 5 questions, excluding the “does not apply” option, were scored. We recorded responses on a 5-point Likert scale, ranging from “strongly disagree” to “strongly agree”. We calculated scores for both measures by reverse coding two items and averaging the responses (possible range 1–5). A higher score indicates a more favorable attitude towards indoor tobacco-free and cannabis-free policies. The Cronbach’s alpha was 0.72 for the tobacco-free attitudes scale and 0.78 for the cannabis-free attitudes scale.

### 2.4 Explanatory variables

#### 2.4.1 Tobacco and cannabis co-use

We dichotomized tobacco use as light versus heavy based on cigarette consumption (≥10 cigarettes per day) (Vijayaraghavan et al., 2024). Cannabis users who used daily in the past 30 days were considered heavy cannabis users (Caulkins et al., 2020), whereas those who used less than daily were considered light users (Kroon et al., 2020). Using these two binary variables, we created a four-level categorical variable of tobacco and cannabis co-use: light tobacco and light cannabis co-use, light tobacco and heavy cannabis co-use, heavy tobacco and light cannabis co-use, heavy tobacco and heavy cannabis co-use.

#### 2.4.2 Predisposing factors

Predisposing factors included self-reported age, gender identity (man, woman, transgender, non-binary), and race and ethnicity (American Indian/Alaska Native, Asian, Native Hawaiian or Other Pacific Islander, Black or African American, White, Hispanic/Latine, More than one race, Unknown/Not reported).

#### 2.4.3 Enabling factors

We asked participants whether they had ever used any cessation medications (e.g., nicotine replacement therapy, bupropion, and varenicline) or other types of cessation aids (e.g., cessation classes, quitlines). We categorized those who responded affirmatively to using medications or non-medication aids as having used cessation aids. We considered having children in one’s home as an enabling factor, but only one participant in the study sample had children under 12 years living with them, so we did not include this variable in our analysis.

#### 2.4.4 Need factors

We estimated average daily cigarette consumption using the number of days smoked in the past 7 days and the number of cigarettes smoked on each smoking day. We used the Cannabis Use Disorder Identification Test (CUDIT) to identify individuals with problematic cannabis use (possible range 0–40) (Adamson & Sellman, 2003).

We screened for post-traumatic stress disorder (PTSD) symptoms using the Primary Care-Post Traumatic Stress Disorder Screen (PC-PTSD-5), applying a cut-off score of 3 or more to determine a positive screen (Bovin et al., 2021). We screened for anxiety using the Generalized Anxiety Disorder scale (GAD-7), and categorized anxiety as minimal (score 0–4), mild (score 5–9), moderate (score 10–14), or severe (score 15–21) (Spitzer et al., 2006). We used the Center of Epidemiologic Studies Depression Scale (CES-D-10) to screen for depression (cut point > 10) (Andresen et al., 1994). We created an ordinal variable for the number of mental health screens (i.e., screens for a mental health condition), including anxiety, depression, and PTSD (range 0 to 3).

We obtained information on past-30-day substance use for opioids, amphetamines, cocaine, and cannabis, and created an ordinal variable for the total number of these substances used (range 0 to 4). We asked participants whether they or a family member they lived with had unmet subsistence needs (food, utilities, medicine, health care, phone, clothing, child care, or other need) in the past year and calculated the total number of unmet needs (range 0 to 7).

### 2.5 Data analysis

We report descriptive statistics as frequencies and column percentages for categorical variables and means and standard deviations for continuous variables. We used linear mixed models with a random intercept to account for clustering by PSH site to examine the bivariate and multivariable association of attitudes toward tobacco-free policies and cannabis-free policies and predisposing, enabling, and need factors. Among the sub-sample of tobacco and cannabis co-users, we examined attitudes toward tobacco-free and cannabis-free policies with the intensity of tobacco and cannabis co-use, as well as predisposing, enabling, and need factors. All analyses were conducted in SAS version 9.4.

## 3 Results

### 3.1 Predisposing, enabling, and need factors

#### 3.1.1 Predisposing, enabling, and need factors of the whole study sample

Participants’ mean age was 54.5 years (SD 10.7, Table 1). Most participants were male (62.7%), 41.8% identified as Black or African American, 25.0% as White, and 15.5% as Hispanic/Latine. Almost two-thirds (63.5%) of the participants had ever used medications or non-medication aids for smoking cessation. The average daily cigarette consumption was 11.1 (SD 7.5), and the average CUDIT score for cannabis use was 6.4 (SD 7.0). Half of the participants had screened positive on two or more mental health screens, 42.8% reported using two or more substances in the past 30 days, and 25.3% of participants reported having three or more unmet subsistence needs.

**Table 1:** Characteristics of participants grouped by predisposing, enabling, and need factors (N = 400).

**Predisposing characteristics**		
Age (Mean, SD)	54.5	(10.7)
Gender a (N, %)		
Male	251	(62.7)
Female	130	(32.5)
Transgender/non-binary	17	(4.3)
Race and ethnicity b (N, %)		
Black or African American	167	(41.8)
White	100	(25.0)
Hispanic/Latino	62	(15.5)
American Indian/Alaska Native	10	(2.5)
Asian	4	(1.0)
Native Hawaiian or other pacific Islander	4	(1.0)
Multiracial	48	(12.0)
**Enabling factors**		
Ever used medication or non-medication resources for smoking cessation, (N, %)	254	(63.5)
**Need factors**		
Past week average daily cigarette consumption (Mean, SD)	11.1	(7.5)
Cannabis use [CUDIT score] (Mean, SD) c	6.4	(7.0)
Number of positive mental health screens, (N, %) d		
None	118	(29.5)
One	79	(19.8)
Two	100	(25.0)
Three	102	(25.5)
Use of substances in past 30 days, (N, %) e		
Cannabis	257	(64.3)
Cocaine	109	(27.3)
Amphetamines	126	(31.5)
Opioids	79	(19.8)
Number of substances used, (N, %) f		
None	75	(18.8)
Onewo or more	151	(37.8)
Two or more	171	(42.8)
Number of unmet subsistence needs, (N, %) g		
None	147	(36.8)
One	89	(22.3)
Two	63	(15.8)
Three or more	101	(25.3)

SD: Standard deviation; CUDIT: Cannabis Use Disorder Identification Test.

a 2 participants declined to answer.

b 5 participants declined to answer.

c Missing data for 4 participants.

d Missing data for 1 participant; Mental health screens counted were depression, anxiety and post-traumatic stress disorder.

e Missing data for 3 participants for cannabis use, 8 participants for cocaine use, 8 participants for amphetamine use, 10 participants for opioid use.

f Missing data for 3 participants; Use of opioids, amphetamines, cocaine, and cannabis in the past 30 days.

g Unmet subsistence needs recorded were food, utilities, medicine or any health care (medical, dental, mental health, vision), phone, clothing, childcare, other.

#### 3.1.2 Predisposing, enabling, and need characteristics of tobacco and cannabis co-users

Of the 400 participants, 257 co-used tobacco and cannabis. Among the co-users, 26.8% were light tobacco and light cannabis co-users, 17.5% were light tobacco and heavy cannabis co-users, 26.8% were heavy tobacco and light cannabis co-users, and 28.8% were heavy tobacco and heavy cannabis co-users (Table 2).

**Table 2:** Characteristics of tobacco and cannabis co-users by intensity of use grouped by predisposing, enabling, and need factors.

	Overall Sample		Light tobacco, light cannabis		Light tobacco, heavy cannabis		Heavy tobacco, light cannabis		Heavy tobacco, heavy cannabis	
	(N = 257)		(n = 69)		(n = 45)		(n = 69)		(n = 74)	
**Predisposing characteristics**										
Age, Mean (SD)	53.2	(10.8)	56.1	(10.3)	51.4	(11.2)	52.6	(11.2)	51.9	(10.5)
Gender (N, %) a										
Male	174	(67.7)	44	(63.8)	34	(75.6)	50	(72.5)	46	(62.2)
Female	71	(27.6)	22	(31.9)	8	(17.8)	17	(24.6)	24	(32.4)
Transgender/non-binary	10	(3.9)	3	(4.4)	1	(2.2)	2	(2.9)	4	(5.4)
Race/ethnicity (N, %) b										
Black or African American	113	(44.0)	31	(44.9)	20	(44.4)	34	(49.3)	28	(37.8)
White	58	(22.6)	14	(20.3)	10	(22.2)	16	(23.2)	18	(24.3)
Hispanic/Latine	41	(16.0)	13	(18.8)	7	(15.6)	8	(11.6)	13	(17.6)
American Indian/Alaska Native	6	(2.3)	3	(4.4)	0	(0.0)	0	(0.0)	3	(4.1)
Asian	3	(1.2)	1	(1.5)	0	(0.0)	1	(1.5)	1	(1.4)
Native Hawaiian or other pacific Islander	2	(0.8)	1	(1.5)	0	(0.0)	1	(1.5)	0	(0.0)
Multiracial	30	(11.7)	3	(4.4)	8	(17.8)	8	(11.6)	11	(14.9)
**Enabling factors**										
Ever used medication or non-medication resources (N, %) for smoking cessation	157	(61.1)	42	(60.9)	27	(60.0)	44	(63.8)	44	(59.5)
**Need factors**										
Average daily cigarette consumption − Mean (SD)	10.7	(6.8)	5.5	(1.7)	4.8	(1.8)	15.6	(7.0)	14.5	(5.3)
Cannabis use [CUDIT score] c − Mean (SD)	9.6	(6.6)	5.6	(4.6)	11.3	(5.6)	8.1	(5.6)	13.8	(7.1)
Number of positive mental health screens, (N, %) d										
None	69	(26.9)	23	(33.3)	18	(40.0)	15	(21.7)	13	(17.6)
One	54	(21.0)	17	(24.6)	7	(15.6)	18	(26.1)	12	(16.2)
Two	67	(26.1)	13	(18.8)	15	(33.3)	19	(27.5)	20	(27.0)
Three	66	(25.7)	16	(23.2)	5	(11.1)	16	(23.2)	29	(39.2)
Use of substances in past 30 days, (N, %) e										
Cocaine	77	(30.0)	26	(37.7)	7	(15.6)	25	(36.2)	19	(25.7)
Amphetamines	93	(36.2)	18	(26.1)	14	(31.1)	33	(47.8)	28	(37.8)
Opioids	54	(21.0)	12	(17.4)	6	(13.3)	21	(30.4)	15	(20.3)
Number of substances used (N, %) f										
One	110	(42.8)	28	(40.6)	25	(55.6)	24	(34.8)	33	(44.6)
Two or more	147	(57.2)	41	(59.4)	20	(44.4)	45	(65.2)	41	(55.4)
Number of unmet subsistence needs, (N, %) g										
None	96	(37.4)	30	(43.5)	20	(44.4)	19	(27.5)	27	(36.5)
One	52	(20.2)	16	(23.2)	7	(15.6)	15	(21.7)	14	(18.9)
Two	42	(16.3)	10	(14.5)	8	(17.8)	11	(15.9)	13	(17.6)
Three or more	67	(26.1)	6	(8.7)	3	(6.7)	7	(10.1)	9	(12.2)

SD: Standard deviation; CUDIT: Cannabis Use Disorder Identification Test.

a Missing data for 2 participants.

b Missing data for 4 participants.

c Missing data for 1 participant.

d Missing data for 1 participant; Mental health screens counted were depression, anxiety and post-traumatic stress disorder.

e Missing data for 3 participants for cocaine use, 3 participants for amphetamine use, 4 participants for opioid use.

f Use of opioids, amphetamines, cocaine, and cannabis in the past 30 days.

g Unmet subsistence needs recorded were food, utilities, medicine or any health care (medical, dental, mental health, vision), phone, clothing, childcare, other.

The average daily cigarette consumption was 10.7 (SD 6.8), with heavy tobacco users consuming the most cigarettes on average (15.6 for heavy tobacco and light cannabis co-users, and 14.5 for heavy tobacco and heavy cannabis co-users). More than half the co-users screened positive on two or more mental health screens, with 66.2% of heavy tobacco and cannabis co-users screening positive for at least two of the depression, anxiety, or PTSD screens. Over half of the tobacco and cannabis co-users (57.2%) reported using two or more substances in the past 30 days, with cocaine (30%) and amphetamines (36.2%) being the most used substances.

### 3.2 Predisposing, enabling, and need factors associated with attitudes toward tobacco-free policies in the whole sample

In bivariate models, Hispanic/Latine participants (β:0.23, 95% Confidence Interval [CI]:0.004, 0.47) had more favorable attitudes toward tobacco-free policies compared to White participants, though this association was not statistically significant in multivariable analysis (Table 3). Higher average daily cigarette consumption (β:-0.08, 95% CI:-0.13, −0.03), use of cocaine in the past 30 days (β:-0.19, 95% CI:-0.34, −0.03), and having three or more unmet subsistence needs (β:-0.19, 95% CI:-0.38, −0.001) were associated with less favorable attitudes toward tobacco-free policies compared to their counterparts.

**Table 3:** Linear mixed models of the association of predisposing, enabling, and need factors and attitudes toward tobacco-free policies in the whole sample.

	Bivariate analyses Unadjusted β-coefficient (95% Confidence Interval)		Multivariable analysis (N = 390) a Adjusted β-coefficient (95% Confidence Interval)	
Age b	0.002	(−0.03, 0.04)	−0.01	(−0.05, 0.03)
Gender (ref: Male)				
Non-male c	0.03	(−0.12, 0.18)	0.02	(−0.13, 0.17)
Race/ethnicity (ref: White)				
Black	0.09	(−0.09, 0.27)	0.10	(−0.097, 0.30)
Hispanic/ Latino	0.23	(0.004, 0.47)*	0.22	(−0.02, 0.45)
Other d	0.05	(−0.17, 0.27)	0.04	(−0.18, 0.27)
Ever used medication or non-medication resources for smoking cessation	0.05	(−0.10, 0.20)	0.09	(−0.06, 0.24)
Average daily cigarette consumption e	−0.08	(−0.13, −0.03)*	−0.07	(−0.12, −0.02)*
Cannabis use [CUDIT score]	−0.01	(−0.02, 0.002)	−0.01	(−0.03, 0.002)
Number of positive mental health screens f (Ref: None)				
One	0.2	(−0.01, 0.41)	0.24	(0.03, 0.45)*
Two	0.03	(−0.17, 0.22)	0.13	(−0.07, 0.34)
Three	0.01	(−0.18, 0.21)	0.12	(−0.10, 0.33)
Use of substances in past 30 days, (N, %) g				
Cannabis	−0.02	(−0.17, 0.13)	0.07	(−0.13, 0.26)
Cocaine	−0.19	(−0.34, −0.03)*	−0.20	(−0.37, −0.04)*
Amphetamines	−0.15	(−0.31, 0.003)	−0.13	(−0.31, 0.05)
Opioids	0.008	(−0.17, 0.19)	0.14	(−0.06, 0.34)
Number of unmet needs h (Ref: no unmet needs)				
One	0.01	(−0.18, 0.20)	−0.01	(−0.20, 0.19)
Two	−0.18	(−0.40, 0.03)	−0.24	(−0.47, −0.02)*
Three or more	−0.19	(−0.38, −0.001)*	−0.23	(−0.43, −0.02)*
				

a Score range for attitudes toward tobacco-free policies is 1–5; 10 participants from the multivariable model due to missing values on one or more independent variables.

b Age scaled to 5-year units for interpretability.

c Includes female, transgender and non-binary.

d Includes Asian, Native Hawaiian or other Pacific Islander, Multiracial, and American Indian/ Alaska Native.

e Cigarettes per day scaled to 5-unit increase for interpretability.

f Mental health screens counted were depression, anxiety and post-traumatic stress disorder.

g Missing data for 3 participants for cannabis use, 8 participants for cocaine use, 8 participants for amphetamine use, 10 participants for opioid use.

h Unmet subsistence needs recorded were food, utilities, medicine or any health care (medical, dental, mental health, vision), phone, clothing, childcare, other CUDIT: Cannabis Use Disorder Identification Test.

* p < 0.05.

In the multivariable analysis (Table 3), having one positive mental health screen (adjusted β:0.24, 95% CI:0.03, 0.45) compared to none was associated with more favorable attitudes toward tobacco-free policies. Using cocaine in the past 30 days (adjusted β:-0.20, 95% CI:-0.37, −0.04) and having three or more unmet subsistence needs compared to no unmet needs (adjusted β:-0.23, 95% CI:-0.43, −0.02) were associated with less favorable attitudes towards tobacco-free policies.

### 3.3 Predisposing, enabling, and need factors associated with attitudes toward tobacco-free policies and cannabis-free policies among tobacco and cannabis co-users

#### 3.3.1 Attitudes toward tobacco-free policies among tobacco and cannabis co-users

In bivariate models (Table 4), having heavy tobacco and heavy cannabis co-use compared to light tobacco and light cannabis co-use (β:-0.28, 95% CI:-0.52, −0.04) was associated with less favorable attitudes toward tobacco-free policies. However, having one positive mental health screen compared to none was associated with more favorable attitudes toward tobacco-free policies (β:0.34, 95% CI:0.08, 0.60). Results were similar in the multivariable analysis, where intensity of tobacco and cannabis co-use and mental health screens were associated with attitudes toward tobacco-free policies. Use of cocaine in the past 30 days was associated with less favorable attitudes toward tobacco-free policies (β:-0.21, 95% CI:-0.41, −0.02) in bivariate analysis, but not in multivariable analysis.

**Table 4:** Linear mixed models of attitudes toward tobacco-free policies and cannabis-free policies with predisposing characteristics, enabling resources, and need factors among tobacco and cannabis co-users.

	Attitudes towards tobacco-free policies				Attitudes towards cannabis-free policies			
	Bivariate analyses Unadjusted β-coefficient (95% Confidence Interval)		Multivariable analysis^a^ (N = 253) Adjusted β-coefficient (95% Confidence Interval)		Bivariate analyses Unadjusted β-coefficient (95% Confidence Interval)		Multivariable analysis b (N = 246) Adjusted β-coefficient (95% Confidence Interval)	
Tobacco and cannabis co-use categories(Ref: light tobacco, light cannabis)								
Light tobacco, heavy cannabis	0.08	(−0.19, 0.36)	0.06	(−0.22, 0.35)	−0.31	(−0.58, −0.04)*	−0.32	(−0.6, −0.04)*
Heavy tobacco, light cannabis	−0.1	(−0.35, 0.15)	−0.12	(−0.37, 0.13)	−0.02	(−0.27, 0.23)	−0.02	(−0.27, 0.23)
Heavy tobacco, heavy cannabis	−0.28	(−0.52, −0.04)*	−0.32	(−0.56, −0.07)*	−0.59	(−0.83, −0.35)**	−0.58	(−0.82, −0.35)*
Age c	−0.02	(−0.06, 0.02)	−0.02	(−0.07, 0.03)	0.04	(−0.01, 0.08)	0.001	(−0.05, 0.05)
Gender (Ref: Male)								
Non-male d	0.04	(−0.16, 0.23)	0.03	(−0.17, 0.23)	−0.22	(−0.42, −0.02)*	−0.21	(−0.41, −0.01)*
Race/ethnicity (Ref: White)								
Black	−0.06	(−0.30, 0.17)	0.003	(−0.26, 0.27)	0.2	(−0.04, 0.44)	0.10	(−0.16, 0.36)
Hispanic/ Latino	0.15	(−0.14, 0.45)	0.21	(−0.1, 0.52)	0.31	(0.003, 0.61)*	0.29	(−0.01, 0.59)
Other e	0.01	(−0.28, 0.30)	0.05	(−0.23, 0.34)	0.01	(−0.28, 0.31)	0.04	(−0.25, 0.32)
Ever used medication or non-medication resources for smoking cessation	0.03	(−0.15, 0.22)	0.07	(−0.12, 0.26)	−0.21	(−0.40, −0.02)*	−0.16	(−0.35, 0.02)
Number of positive mental health screens f (Ref: None)								
One	0.34	(0.08, 0.60)*	0.38	(0.11, 0.64)*	0.06	(−0.21, 0.33)	0.02	(−0.24, 0.28)
Two	0.14	(−0.10, 0.39)	0.17	(−0.1, 0.43)	−0.09	(−0.35, 0.17)	0.005	(−0.25, 0.26)
Three	0.06	(−0.19, 0.31)	0.11	(−0.18, 0.4)	−0.12	(−0.38, 0.14)	−0.0001	(−0.28, 0.28)
Use of substances in past 30 days g								
Cocaine	−0.21	(−0.41, −0.02)*	−0.19	(−0.4, 0.02)	−0.02	(−0.22, 0.18)	−0.07	(−0.27, 0.13)
Amphetamines	−0.1	(−0.29, 0.09)	−0.21	(−0.44, 0.01)	−0.07	(−0.27, 0.13)	−0.07	(−0.29, 0.14)
Opioids	0.11	(−0.12, 0.33)	0.25	(−0.01, 0.52)	−0.12	(−0.35, 0.11)	−0.04	(−0.29, 0.22)
Number of unmet subsistence needs (Ref: No unmet needs) h								
One	0.12	(−0.13, 0.36)	0.09	(−0.16, 0.34)	0.11	(−0.15, 0.37)	0.11	(−0.14, 0.36)
Two	0.09	(−0.18, 0.36)	0.01	(−0.28, 0.3)	−0.005	(−0.28, 0.27)	0.04	(−0.24, 0.32)
Three or more	−0.09	(−0.32, 0.15)	−0.12	(−0.38, 0.14)	−0.05	(−0.29, 0.19)	0.03	(−0.22, 0.28)

a Score range for attitudes toward tobacco-free policies is 1–5; 4 participants dropped from model due to missing values on one or more independent variables.

b Score range for attitudes toward cannabis-free policies is 1–5; 11 participants dropped from model due to missing values on one or more independent variables.

c Age scaled to 5-year units for interpretability.

d Includes female, transgender and non-binary.

e Includes Asian, Native Hawaiian or other Pacific Islander, Multiracial, and American Indian/ Alaska Native.

f Mental health screens counted were depression, anxiety and post-traumatic stress disorder.

g Missing data for 3 participants for cocaine use, 3 participants for amphetamine use, 4 participants for opioid use in tobacco-free attitudes bivariate models; Missing data for 10 participants for cocaine use, 10 participants for amphetamine use, 11 participants for opioid use in cannabis-free attitudes bivariate models.

h Unmet subsistence needs recorded were food, utilities, medicine or any health care (medical, dental, mental health, vision), phone, clothing, childcare, other.

* p < 0.05,

** p < 0.001.

#### 3.3.2 Attitudes toward cannabis-free policies among tobacco and cannabis co-users

In bivariate analysis (Table 4), the light tobacco and heavy cannabis co-users (β:-0.31; 95% CI:-0.58, −0.04) and the heavy tobacco and heavy cannabis co-users (β:-0.59; 95% CI:-0.83, −0.35) had less favorable attitudes towards cannabis-free policies compared to light tobacco and light cannabis co-users. Being non-male (adjusted β:-0.22; 95% CI:-0.42,-0.02) was associated with less favorable attitudes towards cannabis-free policies. Results were similar in multivariable analysis. Other substance use in the past 30 days was not associated with attitudes toward cannabis-free policies among co-users.

## 4 Discussion

To our knowledge, this is the first study to examine attitudes toward tobacco-free and cannabis-free policies among formerly homeless adults living in PSH, who have a high prevalence of positive mental health screens and co-occurring substance use. This is also the first study to apply the predisposing, enabling, and need framework to identify modifiable factors associated with attitudes towards indoor tobacco-free and cannabis-free policies. About two-thirds of the sample (64.3%) co-used tobacco and cannabis, about half had one positive mental health screen, half reported non-tobacco substance use, and more than half had an unmet subsistence need. Cocaine and amphetamines were the most common forms of other substances used in this sample. Improving attitudes towards smoke-free policies in this sample of high-intensity users of tobacco and cannabis may support smoke-free policy implementation and reduce tobacco use behaviors and SHS exposure from tobacco and cannabis co-use among residents in multi-unit housing (Mills et al., 2011). Our findings underscore the need for considering the intensity of tobacco and cannabis co-use and co-occurring substance use when implementing smoke-free policies in multi-unit PSH.

Among need factors, cocaine use in the past 30 days was associated with less favorable attitudes toward tobacco-free policies compared to no cocaine use. A prior study found a bidirectional association between the use of cocaine and cigarette smoking, such that the use of each product increased the craving, intensity, and pleasurable effects of the other product (Brewer et al., 2013 May). Given the significant cardiovascular and respiratory-related harms associated with the ongoing use of smoked cocaine among tobacco users (Winhusen et al., 2020 Sep), our study has implications for integrating treatment for substance use with tobacco use among adults living in PSH.

Among need factors, a greater number of unmet subsistence needs was associated with less favorable attitudes towards tobacco-free policies. In a study of Black/African American women living in low-resource areas, women who had difficulty meeting basic needs were significantly less likely to have a complete ban on the use of all tobacco products in their home compared to women who said they lived comfortably (Jones et al., 2022). Tobacco use is also associated with food insecurity, siphoning off available income from basic needs like food (Gu et al., 2023). Our findings underscore the importance of understanding the role of financial and housing security, as well as the presence of unmet subsistence needs, in residents’ attitudes toward tobacco-free policies. Interventions that alleviate unmet subsistence needs as part of tobacco treatment interventions (Kathuria et al., 2022, Park et al., 2025) are needed, particularly given that tobacco may be used to cope with stressors associated with poverty.

We explored only one enabling factor—use of medication or non-medication tobacco cessation aids—and this factor was not associated with attitudes toward tobacco-free or cannabis-free policies in multivariable models. Other enabling factors, such as having young children in one’s home, less relevant to our study sample of mostly single adults, could influence attitudes toward tobacco- or cannabis-free policies (Mills et al., 2009).

Having one positive mental health screen compared to none was associated with more favorable attitudes toward tobacco-free policies among tobacco users and tobacco and cannabis co-users. A prior study among PSH managers and staff showed that the severity of mental health conditions among residents may be associated with less adherence to restrictions on indoor smoking, and severe symptoms of depression or agoraphobia may be a barrier to adhering to an indoor smoking rule (Alizaga et al., 2020). There has long been a prevailing tobacco-industry-supported myth that tobacco use may be used to alleviate mental health symptoms (Prochaska et al., 2008). Findings from our study warrant further exploration on the role of positive mental health screens and mental health diagnoses on attitudes toward tobacco-free and cannabis-free policies.

In 2021, the prevalence of tobacco and cannabis co-use was 6.38% in the US population, with co-use increasing across all socioeconomic strata between 2002 and 2019 (Rubenstein et al., 2024). Treatment for tobacco and cannabis co-use and restricting their use indoors is challenging as people have different perceptions of risk and harm from co-use, including the intensity of co-use (Popova et al., 2017), perceptions of interrelatedness (i.e., temporally concurrent use), and the impact of one substance on the other (Akbar et al., 2019). Understanding the relatedness of tobacco and cannabis co-use among PSH residents, as well as the intensity of co-use, may help in creating tailored strategies to increase favorable attitudes toward tobacco and cannabis-free policies and reduce co-use behaviors.

Non-male co-users of tobacco and cannabis had less favorable attitudes toward cannabis-free policies than male co-users, even after adjusting for intensity of tobacco and cannabis co-use. There is increasing research on the negative role that cannabis plays in diseases that disproportionately affect women, including anxiety, depression, post-traumatic stress disorder, and other conditions (Gräfe et al., 2023). Our findings highlight the need to better understand tobacco and cannabis co-use behaviors among non-male residents in PSH.

Our study had several limitations. Findings are based on a large sample of PSH in the San Francisco Bay Area, and they may not be generalizable regionally or nationally. The cross-sectional analysis limits causal inferences. Self-reports may be subject to recall bias and social desirability bias. We did not explore indoor use of vapes or other combustible tobacco, like cigars. We did not conduct an exploratory factor analysis for the attitudes scales; however, the tobacco-free attitudes scale performed similarly in our previous studies of adults living in permanent supportive housing (Durazo et al., 2020, Petersen et al., 2018, Petersen et al., 2020, Vijayaraghavan and Pierce, 2015).

Our study among a large diverse sample of residents living in PSH highlights that a substantial proportion of PSH residents who smoke cigarettes, concurrently use tobacco and cannabis and other substances. The high rates of co-use highlight the need for interventions that provide education on the harms of indoor use of tobacco and cannabis products, resources for cessation, as well as integrated interventions for substance use. Such interventions may improve attitudes toward smoke-free policies and, in turn, future adoption of smoke-free policies. Addressing unmet subsistence needs will be crucial to implementing smoke-free policies for residents in PSH.

**Role of funding source**.

This work was supported by the National Cancer Institute at the National Institute of Health (R37CA248448, PI: Vijayaraghavan). N.N was also supported by the National Institute on Minority Health and Health Disparities (R01MD016898, PI:Vijayaraghavan) and the University of California, San Francisco Global Scholars Program.

## CRediT authorship contribution statement

**Narges Neyazi:** Writing – original draft, Methodology, Conceptualization. **Deepalika Chakravarty:** Writing – review & editing, Investigation, Formal analysis, Data curation, Conceptualization. **Fan Xia:** Writing – review & editing, Visualization, Validation, Methodology, Formal analysis. **Mark R. Hawes:** . **Wendy Max:** Writing – review & editing, Conceptualization. **Margot Kushel:** Writing – review & editing. **Maya Vijayaraghavan:** Writing – review & editing, Writing – original draft, Supervision, Project administration, Methodology, Funding acquisition, Conceptualization.

## Declaration of competing interest

The authors declare that they have no known competing financial interests or personal relationships that could have appeared to influence the work reported in this paper.

## Data Availability

Data will be made available on request.
